# Validation of a Patient-Reported Outcome Measure for Moist Desquamation among Breast Radiotherapy Patients

**DOI:** 10.3390/curroncol29070376

**Published:** 2022-07-07

**Authors:** Cheryl Duzenli, Elisa K. Chan, Theodora Koulis, Sheri Grahame, Joel Singer, David Morris, Josslynn Spence, Terry Lee, Levi Burns, Robert A. Olson

**Affiliations:** 1 Department of Medical Physics, BC Cancer, Vancouver, BC V5Z 4E6, Canada; 2 Department of Surgery, Division of Radiation Oncology and Developmental Radiotherapeutics, University of British Columbia, Vancouver, BC V5Z 1M9, Canada; elisa.chan@bccancer.bc.ca (E.K.C.); theodora.koulis@bccancer.bc.ca (T.K.); rolson2@bccancer.bc.ca (R.A.O.); 3 Department of Physics and Astronomy, University of British Columbia, Vancouver, BC V6T 1Z1, Canada; 4 Department of Radiation Oncology, BC Cancer, Vancouver, BC V5Z 4E6, Canada; 5 Department of Radiation Oncology, BC Cancer, Victoria, BC V8R 6V5, Canada; 6 Department of Radiation Therapy, BC Cancer, Vancouver, BC V5Z 4E6, Canada; slomas-02@bccancer.bc.ca; 7 School of Population and Public Health, University of British Columbia, Vancouver, BC V6T 1Z3, Canada; jsinger@hivnet.ubc.ca; 8 Centre for Health Evaluation and Outcome Sciences, St. Paul’s Hospital, Vancouver, BC V6Z 1Y6, Canada; tlee@hivnet.ubc.ca; 9 Department of Radiation Therapy, BC Cancer, Prince George, BC V2M 7E9, Canada; dmorris2@bccancer.bc.ca (D.M.); josslynn.spence@bccancer.bc.ca (J.S.); 10 Michael G. DeGroote School of Medicine, McMaster University, Hamilton, ON L8S 4L8, Canada; levi.burns@medportal.ca; 11 Department of Radiation Oncology, BC Cancer, Prince George, BC V2M 7E9, Canada

**Keywords:** breast cancer, breast radiotherapy, skin toxicity, moist desquamation, patient-reported outcomes, radiation dermatitis

## Abstract

There has been an increasing interest in patient-reported outcome (PRO) measures in both the clinical and research settings to improve the quality of life among patients and to identify when clinical intervention may be needed. The primary purpose of this prospective study was to validate an acute breast skin toxicity PRO measure across a broad sample of patient body types undergoing radiation therapy. Between August 2018 and September 2019, 134 women undergoing adjuvant breast radiotherapy (RT) consented to completing serial PRO measures both during and post-RT treatment and to having their skin assessed by trained trial radiation therapists. There was high patient compliance, with 124 patients (92.5%) returning to the clinic post-RT for at least one staff skin assessment. Rates of moist desquamation (MD) in the infra-mammary fold (IMF) by PRO were compared with skin assessments completed by trial radiation therapists. There was high sensitivity (86.5%) and good specificity (79.4%) between PRO and staff-reported presence of MD in the IMF, and there was a moderate correlation between the peak severity of the MD reported by PRO and assessed by staff (rho = 0.61, *p* < 0.001). This prospective study validates a new PRO measure to monitor the presence of MD in the IMF among women receiving breast RT.

## 1. Introduction

The prospective outcomes and support initiative (POSI) is a program designed to develop, collect, and use patient-reported outcomes (PROs) to guide clinical care, drive quality improvement, and empower population-based research in British Columbia, Canada [1]. To date, POSI has collected and used PROs on over 136,000 patient assessments for more than 39,500 different patients, having a direct impact on immediate clinical care and enabling innovative research [2].

Interest in the use of PRO measures to improve clinical care has been increasing. Basch et al. [3] have shown that regular symptom monitoring with PRO measures during systemic treatment can improve quality of life, decrease the rate of hospitalizations and emergency room admissions, and lead to improvement in quality-adjusted survival. Newer clinical trials are also developing and incorporating PROs and quality of life as outcome measures, although more commonly to measure late effects [4,5,6]. A recent meta-analysis evaluated the extent of discordance between clinician-reported outcomes and PROs for radiation dermatitis [7], with very few studies evaluating acute skin toxicity.

Breast cancer patients receiving radiotherapy (RT) are educated to expect skin toxicity and are directed to contact the nursing line if they develop symptoms that require medical attention. Data on breast skin toxicity are not systematically collected as part of routine practice, and PRO instruments have not yet been made widely available for breast RT patients. As a result, there are only modest data on the rates of patients experiencing severe skin toxicity during or immediately after RT treatment and even less on how this impacts their quality of life. It is important to have a baseline understanding of toxicity and then to be able to track if and how the introduction of new technology and advances in breast RT techniques impact the patient experience over time. 

Up to 40% of women receiving adjuvant RT are expected to develop some moist desquamation (MD), a painful and worrisome radiation skin reaction observed when radiation dose exceeds the survival tolerance of cells in the basal layer [8,9,10]. A previous BC Cancer randomized trial to investigate potential advantages of silver leaf nylon dressing to promote wound healing found that 38% of breast RT patients with D cup or larger, or with skin folds 1 cm or deeper, experienced MD at our institution [11]. This is consistent with a recent randomized controlled trial of prone breast RT for a similar patient population [8]. Skin-sparing techniques such as partial breast irradiation and hypo-fractionated breast RT treatment schedules have reported lower rates of acute skin toxicity in intact breast RT [8,12,13]. Other interventions such as a novel carbon-fiber breast support to remove infra-mammary folds (IMFs) are also under investigation for reduction in MD rates [14]. Patients who receive RT post-mastectomy also experience high rates of skin toxicity [10].

High-grade skin toxicity, such as MD, generally peaks 10 to 14 days after RT completion for conventional fractionation schemes. In order to ultimately bring PROs into routine clinical practice to support patients in the post-RT acute reaction phase, a PRO tool would ideally be used one- and two weeks post-RT completion. This poses a challenge for assessment and intervention as patients are generally not in clinic at that time. Remote monitoring of symptoms using electronic PRO instruments is still in the development and pilot stage [15]. We investigated a breast PRO questionnaire designed for patients to report their experience with acute radiation-induced skin toxicity.

The primary purpose of this prospective study was to validate an acute breast skin toxicity PRO questionnaire for use in routine clinical care and research across a broad sample of patient body types undergoing RT.

Specific objectives included: (1) to establish correlations between patient-reported acute skin toxicity and staff assessments of skin toxicity, with a particular focus on MD in the IMF; (2) to establish estimates of current rates of MD across our breast RT population according to known risk factors including body mass index (BMI) greater than 25, IMFs (more than 1 cm deep), and cup size D and larger.

## 2. Materials and Methods

### 2.1. Inclusion Criteria

All patients older than 18 years of age receiving adjuvant whole breast RT and post-mastectomy RT for breast cancer were eligible to participate. Patients requiring a boost following or prior to tangential irradiation were eligible for the study, as well as those requiring locoregional RT.

### 2.2. Exclusion Criteria

Patients were ineligible if they were not expected to be able to complete the POSI study questionnaire at the designated time points post-RT. Patients who could complete the questionnaire but did not attend the skin assessments post-RT were still eligible. The questionnaire was only available in English, but patients were eligible if a translator or family member fluent in English was available for translation.

### 2.3. Recruitment and Consent

Breast RT patients were offered participation in this study over a one-year period between August 2018 and September 2019 at three RT treatment centers. Trial radiation therapists obtained consent at the time of the computed tomography (CT) simulation appointment. Patients consenting to this trial were asked to come 10 min early or stay after treatment to complete the POSI study questionnaire by electronic tablet and meet with the trial radiation therapist for skin assessment at baseline and during the penultimate week and last week of RT. As an optional part of the study, patients were asked if they were willing and reasonably confident that they would be able to return to the clinic for skin assessments one- and two weeks post-RT. Patients were also asked again at the time of their last RT treatment appointment. Those showing interest had appointments scheduled with the trial radiation therapist. POSI data from all patients contributed to the study of whether or not the patient returned for skin assessments post-RT.

### 2.4. Baseline Data

The following baseline data were collected on all patients at CT appointment and during the treatment planning process: age, smoking status, breast cup and bra band size, BMI, and measured IMF depth (in treatment position). IMF depth was measured using a ruler placed in the fold at the deepest point with the patient in the treatment position at CT simulation. The treatment technique and planned dose and fractionation were captured during treatment planning. Previous or current use of chemotherapy and hormonal therapy was also recorded.

### 2.5. Treatment

Treatment prescriptions included 42.5 Gy in 16 fractions and 50 Gy or 50.4 Gy in 25 or 28 fractions. Opposed tangential fields using field-in-field forward planning and step and shoot intensity modulated radiation therapy (IMRT) were used, with single or combined beam energies of 6 MV, 10 MV, and 15 MV. The majority of treatments used 6 MV or 6 MV and 10 MV combined.

### 2.6. PRO Data Collection

The 12-question POSI questionnaire (available in Supplementary Materials) asked patients about skin care product use, including topical steroids and antibiotics, fatigue, pain, pain medication, and degree of skin symptoms, including redness or darkness, itchiness, flakiness or peeling within the last seven days. Since the primary focus of this study was to identify MD, patients were also asked if any open skin occurred within the past seven days and to specify locations where areas of open skin were located as well as the size of the open area. In addition, patients were asked how much their skin condition as a result of treatment interfered with their sleep and activities. Radiation oncologists, trial radiation therapists, and patients provided input to the design of this questionnaire.

The POSI questionnaire was completed at baseline and up to four subsequent time points: (1) penultimate week of treatment, (2) last week of treatment, (3) one week after treatment, and (4) two weeks after treatment. POSI questionnaire data were collected from patients during clinic appointments by means of electronic tablets. All patients were offered access to an electronic or paper version of the questionnaire at one- and two weeks post-RT, in addition to being offered the opportunity to complete this by electronic tablet if they returned to the clinic for skin assessments at these two post-RT time points. As some patients received an additional boost to the tumor bed, the timing of one- and two weeks post-treatment was defined as post-tangential field treatment for this study.

### 2.7. Patient Body Type Characterization

Patients were categorized into five groups as follows: (1) BMI below 25 and IMF less than 1 cm, cup size from A to C; (2) BMI 25 or greater and IMF 1 cm or greater, cup size D or greater; (3) BMI below 25 and IMF 1 cm or greater, cup size D or greater; (4) BMI 25 or greater and IMF less than 1 cm, any cup size; (5) post-mastectomy. Group 1 was considered to be at the lowest risk for MD, while group 2 was considered to be at the highest risk for MD [9,10]. Participants with BMI, IMF, and cup size not strictly fitting these categories were grouped according to BMI and IMF status.

### 2.8. Sample Size

The expectation was to obtain skin assessments one- and two weeks post-RT in addition to POSI questionnaire data on 10–20% of all breast RT patients seen in the clinic during the course of the study. The study was planned to run for one year or until at least 15 patients in each of groups 1 and 2 had been clinically assessed one- and two weeks post-RT in addition to completing POSI. By including a minimum of 15 patients in both the highest risk group (group 2) and lowest risk group (group 1), we aimed to detect a difference between the expected rate of MD of 40% in the highest risk group compared to 0% in the lowest risk group, using a two-tailed alpha of 5%, and a power of 80% for a Fisher’s exact test.

### 2.9. Skin Assessments

**Figure 1:**
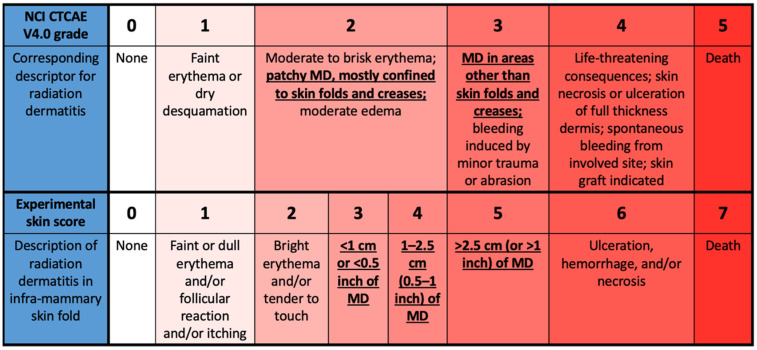
Modified moist desquamation (MD) scale with comparison to NCI CTCAE V4.0 grading. The differences in definition of MD between the two scales are emphasized, including the presence of MD in both grades 2 and 3 of the NCI CTCAE criteria.

Trial radiation therapists scored the degree of skin change using established criteria, National Cancer Institute (NCI) Common Terminology Criteria for Adverse Events (CTCAE) V4.0, at each clinic visit while on RT for all patients and at one- and two weeks post-tangential RT for those patients who chose to return. The NCI CTCAE criteria are not specifically designed to distinguish the presence or absence of MD since different degrees of MD are included in both grade 2 (patchy MD primarily in skin folds) and grade 3 (MD outside skin folds) on this scale. Thus, a modified scale expanding on the NCI CTCAE criteria was used to include MD occurring in skin folds and to quantify the size of the area affected, adapted from Wright et al. [10]. The modified MD scale is shown in Figure 1.

### 2.10. Skin Care

Patients were advised to apply a water-based moisturizer daily starting at the beginning of RT treatment. At the weekly assessment with the physician, further recommendations to manage acute radiation dermatitis (saline compress, topical corticosteroid, topical antibiotic, etc.) were made as needed.

### 2.11. Data Analysis

Descriptive statistics were used to describe the patient population. For each POSI question with a 0–3 severity score, the peak score (highest score across the five time points) was tested for correlation with the peak staff-reported MD score and the peak POSI open skin under the breast score using the Spearman correlation coefficient across the aggregate patient sample. A Spearman’s rho correlation coefficient of 0.6 or greater was considered to signify a good relationship. Sensitivity and specificity of the patient response of “Yes” to “open skin under the breast” from the POSI questionnaire versus the staff-assessed presence of MD in the skin fold was tested using a 2 × 2 table.

Univariate and multivariate analyses of POSI outcomes of pain, pain medication, fatigue, anxiety, activity, and sleep interruption versus peak staff-assessed MD or peak POSI open skin score, smoking, chemo, hormone, and radiation dose were performed using logistic regression. All outcome measures were dichotomized for the clinical significance of the problem (score greater than 1 indicating “quite a bit” or “very much” for symptoms, score of “Yes” for the use of the product, or POSI score greater than 0 indicating the presence of open skin).

## 3. Results

### 3.1. Patients

**Figure 2:**
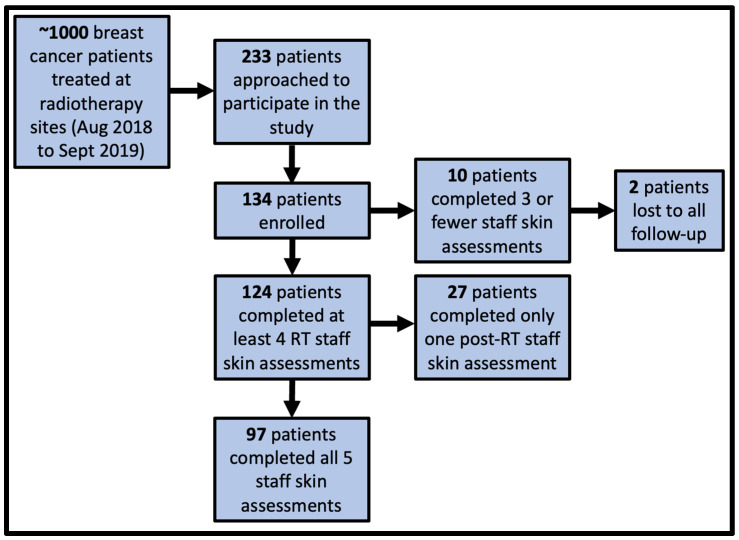
Number of participants who were approached, enrolled, returned to clinic for staff skin assessments, and lost to follow-up. There were three staff skin assessments during the course of radiotherapy (RT) and two post-RT staff skin assessments, resulting in a total of five staff skin assessments for each participant who completed the study.

A total of approximately 1000 breast RT patients were treated during the one-year study period. Participant recruitment and study completion status are shown in Figure 2. In total, 233 patients were approached, and 134 patients were enrolled in the study at three RT treatment centers; 124/134 patients (92.5%) returned to the clinic post-RT for at least one staff skin assessment, and 97/134 (72.4%) patients returned to the clinic for both staff skin assessments and 27/134 (20.1%) returned for one of the post-RT assessments. In total, 10/134 (7.5%) patients did not return to the clinic post-RT for any skin assessments, and two of these ten patients were lost to all follow-ups.

For remote POSI questionnaire completion, 50% of the time, a phone call prompt was conducted because the questionnaire had not been completed within two days of the planned date. Recruitment targets of a minimum of 15 patients in each category were met, except only four patients were recruited in group 3. Baseline patient and treatment characteristics are described in Table 1.

Treatment dose as a function of body type is shown in Table 2. Generally, the higher prescription dose of 50 Gy or 50.4 Gy was used for patients with larger medial-lateral breast separation, resulting in higher percentages of whole breast RT patients with high BMI, large cup size and post-mastectomy patients receiving higher doses, in comparison with group 1 patients. One patient’s dose was reported as 45 Gy, but this is likely to have been a reporting error. One patient prescribed 50 Gy in 25 fractions completed treatment after 24 fractions, receiving 48 Gy.

### 3.2. POSI

As shown in Table 3, 61.2% of patients reported clinically significant (“quite a bit” or “very much”) fatigue in at least one of the questionnaire time points, 51.5% of patients reported clinically significant pain, 29.1% reported clinically significant interruption in daily activities, 29.8% reported clinically significant sleep interruption, and 12.7% reported using clinically significant amounts of pain medication. In total, 68/134 patients (50.7%) required the use of topical steroid cream, and 55 patients (41.0%) required the use of antibiotic cream.

Overall, 52 patients (38.8%) reported MD (open skin) in the IMF for at least one of the POSI questionnaire time points. Patients estimated the peak area of MD to be under 1 cm in 9/134 patients (6.7%), between 1 and 2.5 cm in 12 patients (9.0%), and greater than 2.5 cm in 31 patients (23.1%).

Trial radiation therapists reported MD in 37 patients (27.6%) for at least one of the skin assessment visits. The peak area of MD was measured by staff to be under 1 cm in 16 patients (11.9%), between 1 and 2.5 cm in 4 patients (3.7%), and greater than 2.5 cm in 16 patients (11.9%).

**Figure 3:**
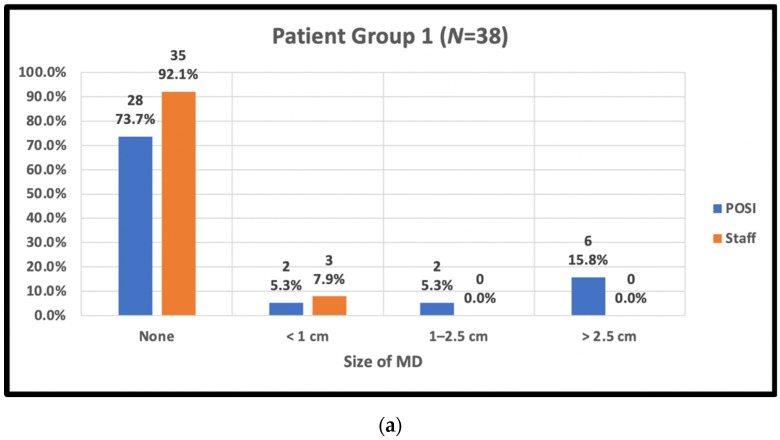

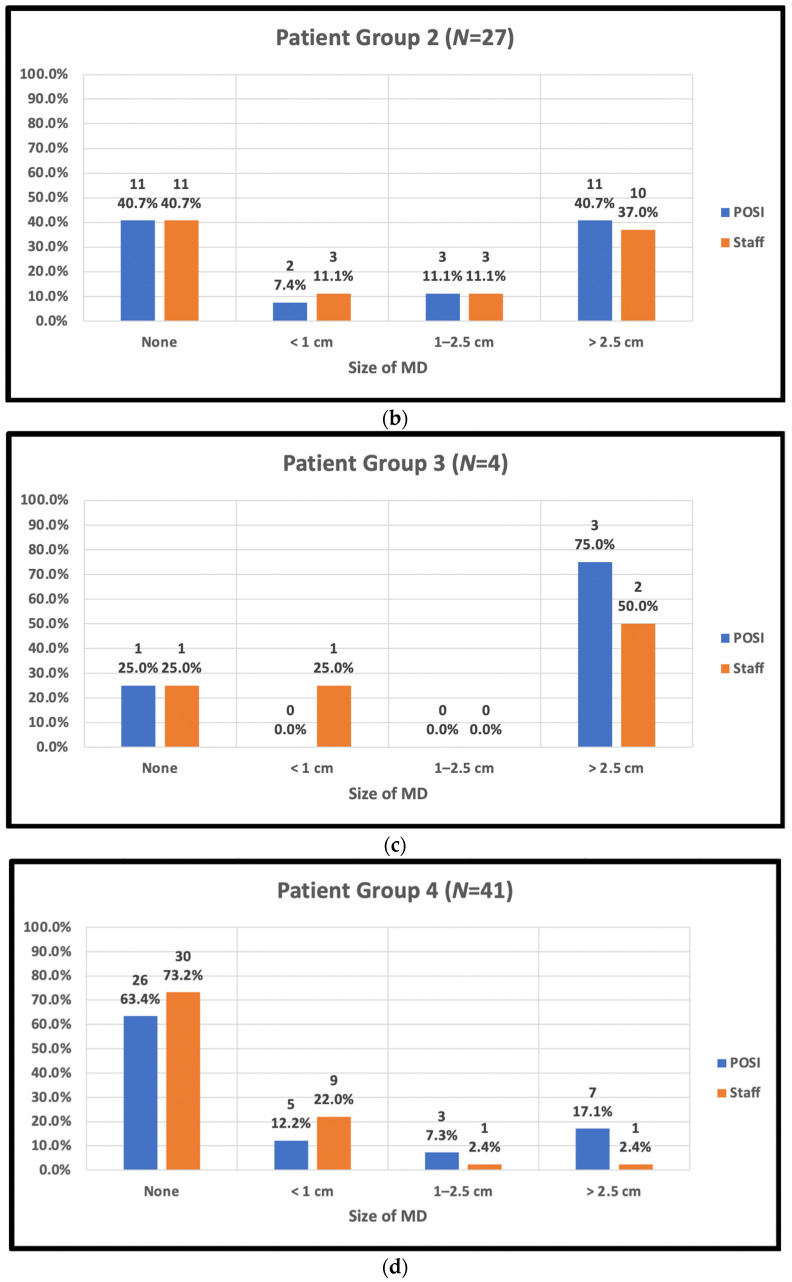

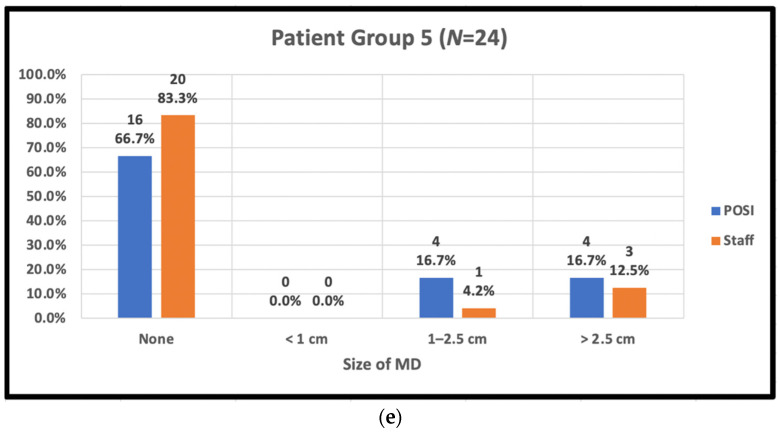
Peak skin moist desquamation (MD) scores reported by staff and in POSI questionnaires by body type. (**a**) Patient body group 1: BMI < 25, cup A–C, IMF < 1 cm; (**b**) patient body group 2: BMI ≥ 25, cup ≥ D, IMF ≥ 1 cm; (**c**) patient body group 3: BMI < 25, Cup ≥ D, IMF ≥ 1 cm; (**d**) patient body group 4: BMI ≥ 25, any cup, IMF < 1 cm; (**e**) patient body group 5: post-mastectomy.

The staff-assessed peak MD area observed by trial radiation therapists and POSI open skin score versus body type is shown in Figure 3.

The peak POSI open skin score had a moderate Spearman correlation with the peak staff skin score that was statistically significant (rho = 0.61, *p* < 0.001). The sensitivity and specificity for POSI open skin to indicate any staff-assessed MD were found to be 86.5% and 79.4%, respectively, as shown in Table 4. Other peak POSI scores for fatigue, pain, pain medication use, activity, and sleep were generally not strongly correlated with the peak staff skin score but showed a somewhat stronger relationship with the POSI peak skin score (rho = 0.36 to 0.43, *p* < 0.001).

On univariate and multivariate logistic regression, patient group 2 (compared to group 1) and total dose of 48 Gy or greater were statistically significantly associated with MD as assessed by staff or by POSI. Whole breast dose of 48 Gy or greater was also correlated with clinically significant pain, fatigue, sleep interruption, and antibiotic use, while group 2 also correlated with antibiotic use. Group 3 had only four patients, so data are not included in this analysis. Details of these results are shown in Table 5.

## 4. Discussion

This prospective study validates the POSI questionnaire to monitor the presence of MD in the IMF among women receiving breast RT. There is high sensitivity (86.5%) and good specificity (79.4%) for open skin in the IMF as assessed by POSI to indicate the presence of MD as assessed by staff. A moderate correlation overall was found between the peak staff skin score and peak POSI open skin score (rho = 0.61, *p* < 0.001).

At this time, there are only a few publications in the literature describing PRO questionnaires for MD. A small study by Montpetit et al. incorporated PRO questions in their study, asking for the presence of open skin, but this was not correlated with a clinical staff assessment [16]. Conversely, Behroozian et al. developed a PRO Likert-type questionnaire to assess RT skin reactions [17]. While blistering and peeling were asked by both patients and staff to determine concordance, the presence of MD was only evaluated by the clinical staff. A recent systematic review of radiation dermatitis assessment tools used in breast cancer described only one PRO measure that evaluated desquamation [18]. However, this PRO measure Skin Toxicity Assessment Tool (STAT), developed by Berthelet et al. [19], had three components. The first component recorded patient and treatment characteristics, the second component was an objective scoring of the skin reaction, which included MD but was only done by staff, while the third component only documented patient-reported symptoms of burning, itchiness, pulling, tenderness, and other complaints. Tagliaferri et al. reviewed cosmetic assessment in brachytherapy for breast cancer and did not report on a PRO measure used to evaluate MD [20]. Shumway et al. recently published a study describing the development of an illustrated scale for acute radiation dermatitis in breast cancer patients [21]. This study evaluated acute radiation dermatitis using PRO, clinician-reported outcomes, and photographic evaluations by clinicians. There was low sensitivity of 38% reported for MD between patient self-reporting and physician assessment by use of photographs, although a high specificity was reported (93%).

The overall rate of staff-reported MD in this study was 27.6% (37/134 patients). When the body types with a higher risk for MD were grouped together (body type groups 2, 3, and 4), the incidence of MD among those patients was 41.7%. Overall rates of MD in a given setting will depend heavily on how the patient population is distributed with respect to various body types. This may explain some of the variation in reported MD rates from study to study [8,13,22].

However, body type alone is not the only factor that determines the incidence of developing MD. We found in this study that the risk of developing MD is strongly dependent on the prescribed dose. On multivariable analysis, patients receiving 48 Gy or greater were more than three times more likely to develop MD compared to patients receiving 45 Gy or less. This is consistent with other published results on the effect of RT fractionation with acute toxicity or body image at the end of treatment [8,13,23]. As well, there are other factors described in the literature, but not analyzed in our study, that have been reported to affect the incidence of MD. These include the location of the hot spot in the IMF during RT planning [24], the prone position [8], and the use of a custom-fit bra or a breast positioning device [25]. The choice of RT technique is also known to impact breast RT outcomes, including MD. Intensity-modulated radiation therapy (IMRT) has been shown to reduce the incidence of MD compared to a wedged technique [9]; patients undergoing VMAT have been shown to be at lower risk of developing radiation dermatitis compared to patients treated with a sliding window IMRT technique [22].

Both patients and staff reported some difficulty with measuring the dimensions of MD areas which may have impacted the overall correlation between peak staff skin score and peak POSI open skin score. Similar issues were commented on in the study by Berthelet et al., who found that while the interobserver reliability for MD was high, there was poor agreement among observers documenting the area of skin reaction (erythema, dry desquamation, and MD) [19]. A less quantitative four-point scale (i.e., “not at all”, “a little bit”, “quite a bit”, and “very much”) may be more practical for the PRO measure going forward. Future work may involve assessing this PRO tool with objective assessments of acute radiation dermatitis, such as spectrophotometry [22], although it remains to be determined whether spectrophotometry is suitable for use in skin folds.

Many patients in this study reported clinically significant (“quite a bit” or “very much”) pain, fatigue, interference of sleep or activity, and even the use of pain medication at some point during their RT treatment or shortly afterward. Total whole breast dose was a strong predictor of pain, fatigue, sleep interruption, and antibiotic use, and peak POSI open skin score was correlated with all symptoms to a lesser degree. Other factors could have contributed to decreased quality of life for patients. Fractionation and race were factors that could play a role in patient-reported breast pain, breast symptoms, or fatigue [26]. Lapen et al. found that discordance between clinician-reported and patient-reported outcomes was greater for psychosocial symptoms or fatigue than for pruritis, pain, or dermatitis, and that discordance increased with time but was in general lower for Black or African American patients [27]. Kishan et al. have suggested that age, baseline sadness and anxiety, and psychiatric or pain-related comorbidities are predictors of patient-reported fatigue [28]. Exercise may also have an effect on fatigue and quality of life [29].

The effect of other treatment factors on quality of life, such as the use and timing of chemotherapy and adjuvant hormonal therapy, as well as the use of boost or locoregional RT, will need to be further studied. Future analysis of peak POSI and staff-reported skin scores in any area of the breast (i.e., not just IMF) may show an improved correlation to these other qualities of life questions. This study reports on the experience of patients receiving external beam breast RT and may not apply to patients receiving brachytherapy. Some studies suggest that patients receiving interstitial partial breast brachytherapy report improved cosmetic outcomes with similar long-term quality of life outcomes compared to external beam RT [30,31].

This prospective study describes the validation of a PRO tool with clinical reported outcomes by trained radiation therapists for evaluating MD. This PRO measure may significantly reduce the burden on trial staff in studies of interventions aimed at reducing acute treatment toxicity, and the problem of inter-observer variability for repeated skin assessments may also be overcome. The shortcoming of the NCI CTCAE categorization of radiation dermatitis grade 2 to include both bright erythema and patchy MD will also be alleviated using this PRO measure. In routine clinical use, this tool could help identify patients experiencing severe acute skin toxicity at home and likely requiring assistance to manage their skin reaction after completion of RT.

However, this study should be interpreted in the context of its strengths and limitations. The distribution of participants by body type is expected to approximately reflect the distribution across the general RT breast population. Figure 2 illustrates how patients were approached and enrolled in the study. Patients were approached as time permitted, but it is unknown what other factors may have influenced which patients were approached and which patients consented to participate. Other limitations to this study include interobserver reliability by the trial radiation therapists was also not studied, the small size of the study as well as small numbers of patients with certain body type characteristics that would limit interpretation of the results among that group of patients.

## 5. Conclusions

This prospective study validates the use of a PRO tool for enabling patients to self-report the presence of MD in a mixed population of body types and breast habitus and correlates well with clinical evaluation for MD by trial radiation therapists. Compliance with this PRO tool is high both during and post-RT treatment. This is a highly accessible means of enabling patients to report acute skin toxicity from breast RT. Future research will investigate the timing and impact of treatment factors on PRO scores, refinement of this POSI questionnaire, and comparison of this questionnaire to objective assessments.

## Figures and Tables

**Table 1:** Baseline patient and treatment characteristics (134 patients total).

Characteristic	Number of Patients	Percentage of Patients
Smoking status		
Never	86	64.2%
Current	6	4.5%
Previous	42	31.3%
Patient body type characterization		
Group 1: BMI < 25, cup size A–C, IMF < 1cm	38	28.4%
Group 2: BMI ≥ 25, cup size ≥ D, IMF ≥ 1cm	27	20.1%
Group 3: BMI < 25, cup size ≥ D, IMF ≥ 1cm	4	3.0%
Group 4: BMI ≥ 25, any cup size, IMF < 1cm	41	30.6%
Group 5: post-mastectomy	24	17.9%
Radiotherapy dose (without boost)		
≤4500 cGy	97	72.4%
≥4800 cGy	37	27.6%
Chemotherapy		
No	79	59.0%
Yes	55	41.0%
Hormone therapy		
No	103	76.9%
Yes	31	23.1%

**Table 2:** Prescribed dose as a function of patient body group (criteria shown in Table 1).

Total Dose (Gy)/Fractions	Patient Body Group				
	Group 1	Group 2	Group 3	Group 4	Group 5
42.5/16	37 (97.4%)	16 (59.3.%)	3 (75.0%)	32 (78.0%)	8 (33.3%)
45	0	0	0	1 (2.4%)	0
48.0/24	0	0	0	0	1 (4.2%)
50.0/25	1 (2.6%)	11 (40.7%)	1 (25.0%)	8 (19.6%)	12 (50.0%)
50.4/28	0	0	0	0	3 (12.5%)

**Table 3:** Summary of peak patient-reported outcome scores at any questionnaire time point.

Score	“Not at All”	“A Little Bit”	“Quite a Bit”	“Very Much”
Peak patient fatigue	7 (5.2%)	45 (33.6%)	61 (45.5%)	21 (15.7%)
Peak patient pain	5 (3.7%)	60 (44.8%)	45 (33.6%)	24 (17.9%)
Peak patient activity	53 (39.6%)	42 (31.3%)	30 (22.4%)	9 (6.7%)
Peak patient sleep	33 (24.6%)	61 (45.5%)	31 (23.1%)	9 (6.7%)
Peak pain medication	67 (50.0%)	50 (37.3%)	12 (9.0%)	5 (3.7%)

**Table 4:** Sensitivity and specificity of POSI open skin to reflect staff assessment of MD. The POSI questionnaire is scored from 0 to 3, with 0 meaning no MD and scores from 1 to 3 indicating some extent of MD.

Peak POSI Score	Peak Staff Skin Score on Modified MD Scale	
	0–2 (*N* = 97)	3–5 (*N* = 37)
0	79% (*N* = 77)	14% (*N* = 5)
1–3	21% (*N* = 20)	86% (*N* = 32)

**Table 5:** Logistic regression results for predictors of MD. Odds ratios are given with 95% lower and upper confidence intervals in parentheses. Results with *p* < 0.05 are marked with an asterisk. Only variables with *p* < 0.1 were included in multivariate regression resulting in some entries for pain or fatigue not being populated.

Variable	Dose ≥ 48 Gy		Body Group 2	
	Univariate	Multivariate	Univariate	Multivariate
Staff skin score	3.2 (1.4–7.3) *	4.4 (1.5–13.1) *	14.6 (3.8–56.2) *	9.6 (2.2–42.1) *
POSI open skin score	2.8 (1.3–6.0) *	3.0 (1.2–7.9) *	3.9 (1.4–11.1) *	2.6 (0.9–8.0)
POSI pain score (“quite a bit” to “very much”)	2.9 (1.3–6.5) *	2.9 (1.3–6.7) *	1.6 (0.6–4.3)	N/A
POSI fatigue score (“quite a bit” to “very much”)	3.5 (1.4–8.7) *	3.5 (1.4–8.7) *	2.0 (0.7–5.8)	N/A
POSI sleep interruption score (“quite a bit” to “very much”)	3.2 (1.4–7.1) *	2.8 (1.1–7.1) *	1.8 (0.6–5.6)	1.2 (0.4–4.0)
Antibiotic use	3.3 (1.5–7.2) *	3.0 (1.2–7.8) *	5.3 (1.8–15.4) *	3.6 (1.2–11.2) *

## Data Availability

The data presented in this study are available on request from the corresponding author. The data are not publicly available due to institutional privacy considerations.

## Supplementary Materials

The 12-question POSI study questionnaire completed by study participants can be downloaded at: https://www.mdpi.com/article/10.3390/curroncol29070376/s1.
