# Supplementary material for: Validation of a Patient-Reported Outcome Measure for Moist Desquamation among Breast Radiotherapy Patients
Source: Curr Oncol. 2022 Jul 7;29(7):4734–47. doi: 10.3390/curroncol29070376 (PMC9325227; doi:10.3390/curroncol29070376)
Supplement: Supplementary file 1 [file curroncol-29-00376-s001.zip › curroncol-1797227-supplementary.pdf]

Prospective Outcomes and Support Initiative (POSI)
Questionnaire for Breast Skin Study

Date: ________________________ (dd/mm/yy) **BCCA#**: ________________________

Please circle or mark one number per line to indicate your response.

Over the past 7 days:

1. **Have you used any skin care products in your breast area?** *(…select none or all that apply)*

None

- ☐ Regular moisturizer (eg: Glaxol®, Lubriderm®, Aveeno®, etc.)
- ☐ Steroid cream (eg: betamethasone or hydrocortisone)
- ☐ Saline soaks
- ☐ Antibiotic cream (eg: Flamazine® or Polysporin®)
- ☐ Other not listed above

	Not At All	A Little bit	Quite a Bit	Very Much
**2. Have you felt tired?**	0	1	2	3
**3. Did you have pain or tenderness in your breast or chest area?**	0	1	2	3
…if you had pain or tenderness **3a. Have you taken medication for the pain in your breast or chest area?**	0	1	2	3

Prospective Outcomes and Support Initiative (POSI) Questionnaire for Breast Skin Study

If you have started or completed your radiation treatments, please complete the remaining questions:

	Over the **past 7 days, in the radiation treatment area**:	Not At All	A Little bit	Quite a Bit	Very Much
4.	Has the skin on your breast or chest area been red or darker than usual?	0	1	2	3
5.	Has the skin on your breast or chest area been itchy?	0	1	2	3
6.	Has the skin on your breast or chest area been dry or flaky?	0	1	2	3
7.	Has the skin on your breast or chest area been peeling?	0	1	2	3
8.	a) Do you have open skin on your breast or chest area?	0	1	2	3
	(If answered 1-3 to 8a)				

b) How much open skin is in the fold under your breast?

- ☐ None
- ☐ Less than1 cm (½ inch)
- ☐ 1 to 2.5 cm (½ inch to1 inch)
- ☐ More than 2.5 cm (> 1 inch)

c) How much open skin is near your armpit area?

- ☐ None
- ☐ Less than 1 cm (½ inch)
- ☐ 1 to 2.5 cm (½ inch to1 inch)
- ☐ More than 2.5 cm (> 1 inch)

d) How much open skin is near the middle of your chest?

- ☐ None
- ☐ Less than 1 cm (½ inch)
- ☐ 1 to 2.5 cm (½ inch to1 inch)
- ☐ More than 2.5 cm (> 1 inch)

e) How much open skin is near the top of your chest/collarbone?

- ☐ None
- ☐ Less than 1 cm (½ inch)
- ☐ 1 to 2.5 cm (½ inch to1 inch)
- ☐ More than 2.5 cm (> 1 inch)

f) How much open skin is in your nipple area?

- ☐ None
- ☐ Less than 1 cm (½ inch)
- ☐ 1 to 2.5 cm (½ inch to1 inch)
- ☐ More than 2.5 cm (> 1 inch)

Not At All	A Little bit	Quite a Bit	Very Much

**9.**	Have the changes in your skin as a result of your breast cancer treatment interfered with your ability to do regular activities such as work, housework, social or recreational activities?	**0**	**1**	**2**	**3**
**10.**	Have the changes in your skin as a result of your breast cancer treatment interfered with your sleep?	**0**	**1**	**2**	**3**
**11.**	Do you feel you have access to the support you need to manage concerns about your skin occurring as a result of treatment?	**Yes or No**			
**12.**	Have you spoken to your Radiation Therapists, Radiation Therapy Nurses or other members of your healthcare team to help you manage concerns about the changes in your skin?	**Yes or No**			

Thank-you for completing this questionnaire
